# FoodOn: a harmonized food ontology to increase global food traceability, quality control and data integration

**DOI:** 10.1038/s41538-018-0032-6

**Published:** 2018-12-18

**Authors:** Damion M. Dooley, Emma J. Griffiths, Gurinder S. Gosal, Pier L. Buttigieg, Robert Hoehndorf, Matthew C. Lange, Lynn M. Schriml, Fiona S. L. Brinkman, William W. L. Hsiao

**Affiliations:** 1 Department of Pathology and Laboratory Medicine, University of British Columbia, Vancouver, BC Canada; 2 Department of Molecular Biology and Biochemistry, Simon Fraser University, Burnaby, BC Canada; 3 Alfred-Wegener-Institut, Helmholtz-Zentrum für Polar- und Meeresforschung, Bremen, Germany; 4 King Abdullah University of Science and Technology, Thuwal, Saudi Arabia; 5 Department of Food Science and Technology, UC Davis, Davis, CA USA; 6 Epidemiology & Public Health, University of Maryland School of Medicine, Baltimore, MD USA; 7 British Columbia Centre for Disease Control Public Health Laboratory, Vancouver, BC Canada; 8 Present Address: Department of Pathology and Laboratory Medicine, University of British Columbia, Vancouver, BC Canada

**Keywords:** Diseases, Plant sciences, Agriculture, Sustainability, Environmental economics

## Abstract

The construction of high capacity data sharing networks to support increasing government and commercial data exchange has highlighted a key roadblock: the content of existing Internet-connected information remains siloed due to a multiplicity of local languages and data dictionaries. This lack of a digital lingua franca is obvious in the domain of human food as materials travel from their wild or farm origin, through processing and distribution chains, to consumers. Well defined, hierarchical vocabulary, connected with logical relationships—in other words, an ontology—is urgently needed to help tackle data harmonization problems that span the domains of food security, safety, quality, production, distribution, and consumer health and convenience. FoodOn (http://foodon.org) is a consortium-driven project to build a comprehensive and easily accessible global farm-to-fork ontology about food, that accurately and consistently describes foods commonly known in cultures from around the world. FoodOn addresses food product terminology gaps and supports food traceability. Focusing on human and domesticated animal food description, FoodOn contains animal and plant food sources, food categories and products, and other facets like preservation processes, contact surfaces, and packaging. Much of FoodOn’s vocabulary comes from transforming LanguaL, a mature and popular food indexing thesaurus, into a World Wide Web Consortium (W3C) OWL Web Ontology Language-formatted vocabulary that provides system interoperability, quality control, and software-driven intelligence. FoodOn compliments other technologies facilitating food traceability, which is becoming critical in this age of increasing globalization of food networks.

## Food Ontology: FoodOn consortium efforts facilitate food traceability

A well-defined food ontology can reduce the barrier of food data harmonization problems world wide. Currently the lack of standard digital vocabulary in food prevents food traceability throughout increasingly global food exchange networks. In this study, Damion Dooley and William Hsiao from the University of British Columbia lead a group of international scientists who give a detailed description of FoodOn, a-consortium-driven project to build a comprehensive food ontology that covers vocabulary from farm to fork. It addresses current gaps in food terminology and focuses on human and domesticated animal food description. It can be foreseen that employing FoodOn vocabulary will speed up traceability of contaminated foods, and ultimately lead to positive economic and human health outcomes.

## Introduction

Digital technology innovation is profoundly affecting many health and economic aspects of food production, distribution, and consumption. The Internet of Things (IoT) is inspiring a vision of network-enabled sensors located in farm environments, shipping containers, factories, retail outlets, and kitchens, all generating data that can be used to increase food quality and guarantee traceability, while reducing resource consumption, cost and wastage. Issues in food safety and security, authenticity and conflicts arising from biocultural trademark protection, and the logistics of local versus multinational food sourcing and distribution are also being analyzed with the help of food-specific datasets and models.^1^ Although agencies benefit from working together on these pan-jurisdictional issues, they face the roadblock that few terminology subdomains have been standardized across sectors and devices (for example, SI weights and measures exist, but reference to measurable qualities like ‘air temperature’ are not standardized internationally). The plethora of food dictionaries keeps food information invisible due to the lack of interoperability, thus impacting the traceability of food, foodborne pathogens, food contaminants, and food quality.

Few internationally-applicable food vocabulary systems have been attempted due to lack of resources and mandates. Technical and language hurdles also discouraged a global repository for cataloging regional foods and their composition. A 1991 International Network of Food Data Systems (INFOODS) paper explains the key factors that led to the setup of regional food composition data centers rather than a single centralized repository.^2^ At the time, the maintenance cost and slow response time of a centralized registry were prohibitive. It was also anticipated that a central registry would be challenged by term ambiguity: “First, it is not possible to be sure that two foods with the same short name in different countries and cultures (or even in different parts of the same country) are so similar that they can be assumed to have the same chemical composition.” The authors also point out the difficulty in achieving cross-cultural and expert consensus about the structure of term hierarchies in use case domains like nutrient content, culinary use, biological taxonomy, and health research with respect to packaging and food additives.

A combination of current internet infrastructure and semantic web technology now make ontology solutions attractive. Ontology provides a formal theory for a domain of inquiry that specifies the meaning of terms within a vocabulary, and consists of a hierarchical taxonomic structure as well as statements (called axioms) about how entities within a domain are related. Appropriate terms can be identified and distinguished by ontology labels and synonyms that include multilingual or region-specific names, as well as globally accessible and unique identifiers and definitions, thus avoiding the use of computationally ambiguous free-text values in food descriptions. For example, in North America “biscuit” refers to a softer “quick bread” (FOODON_03301884), while in Britain it usually means a hard, flat unleavened baked product (FOODON_00002466). Some North American cookies (FOODON_03301585) fall under the British biscuit category too. FoodOn helps resolve such confusion by providing ontology identifiers that yield terms with disambiguating product descriptions.

Ontologies are also capable of accommodating multiple hierarchies often in the form of taxonomies. These hierarchies can act as facets with greater and greater detail/specificity as one navigates deeper into each of the hierarchies within an ontology. For example, a food product can be linked to various international or national food product categories under a “product type” facet, as well as ingredients by way of a hierarchic “food source” facet of plants and animals. Well-designed ontologies reuse as appropriate, terms from other well-established ontologies in order to eliminate duplicates. This enables integration of otherwise disparate ontologies (and their associated data) across domains.^3^ Querying can then occur across federated data, described by a common vocabulary. An ontology enables a vocabulary term to be enhanced with logical axioms that a computer can read and reason over. Automated reasoning software can reveal inferences hidden in stated assumptions, identify potential contradictions or undesired implications, classify new instances based on the instances’ properties, and potentially generate explanations for phenomena such as identifying suspected ingredients in a foodborne disease outbreak scenario.

Incompatibility or ambiguity of basic food reference is a common problem across private and public sectors resolvable by reference to open source consortium-driven ontologies. Launched by the Hsiao Lab, which is affiliated with the University of British Columbia and the British Columbia Centre for Disease Control Public Health Laboratory, the FoodOn project initially targeted the lack of standardized food terminology in public health agency foodborne disease outbreak investigations. A FoodOn founding consortium (http://foodon.org) of mainly OBO Foundry (http://www.obofoundry.org) affiliated members stepped forward to support development since FoodOn fulfilled a gap in that community’s complementary ontologies. The FoodOn consortium promotes a core food description vocabulary that research, consumer and industrial applications can reuse. We foresee standard open controlled vocabularies as the key to enabling IoT-connected food production and processing and distribution transaction systems reducing data exchange costs. The FoodOn value proposition is that a viable standard arises as a conjunction of community-supported and coordinated ontology vocabulary domains—involving plant and animal taxonomy, common names, anatomy, and food description terminology—thus reducing the cost and curation burden that any individual implementer must carry. Industry support of FoodOn through the curation of term submissions will hasten the economic benefit of the emerging “food information superhighway” much as Bluetooth and USB standards reduced the complexity of computer and mobile peripheral communication.

To jumpstart this broader ambition, FoodOn has drawn many of its initial terms from LanguaL, a library science and ontology friendly food classification system consisting of 14 food product description facets including plant or animal food source, chemical additive, preservation or cooking process, packaging, and standard national and international upper-level product type schemes.^4^ LanguaL has evolved steadily from its origin at the Center for Food Safety and Applied Nutrition of the United States Food and Drug Administration (FDA) in the 1970’s. Provided online as a free resource by Danish Food Informatics (http://langual.org), it has been used to index numerous European Union and United States agency databases, including the USDA Nutrient Database for Standard Reference (SR), a food composition database of nutritional data for servings of common and branded food products, and 30 European Food Information Resource (EuroFIR) Food Classification system sanctioned databases.^5,6^ For example, see the Czech Food Composition Database entry for black raw currants (http://www.nutridatabaze.cz/en/food/?id=42#tab-2). In selecting LanguaL, the FoodOn project aims to provide a familiar and professional terminology backbone enhanced with globally accessible ontology technology that can integrate data dictionaries and systems across borders, and describe food products and food production in a consistent and harmonizable way.

## Results

**Fig. 1:**
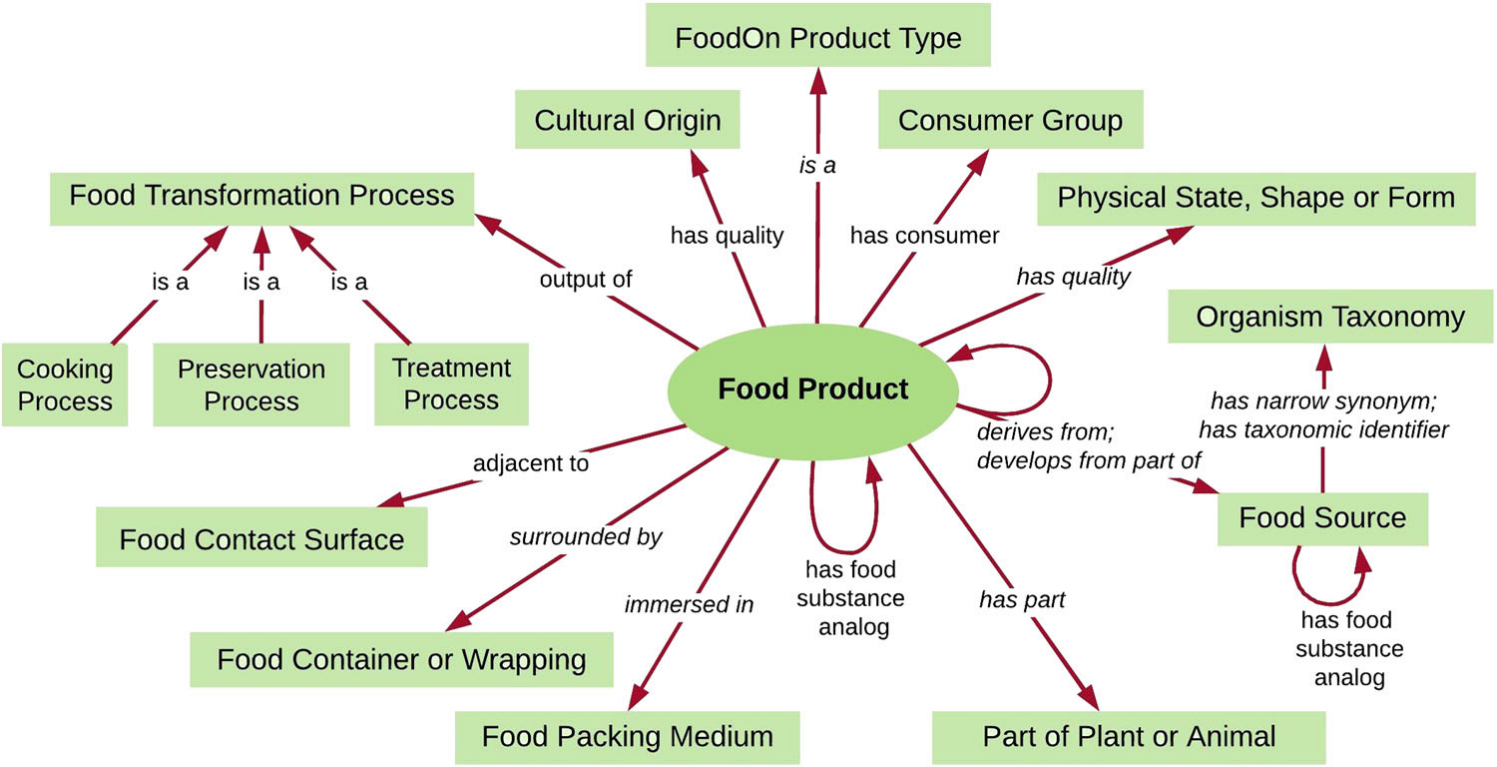
[Food product diagram]. The FoodOn food product scheme derived mainly from LanguaL food description facets, with the addition of ontology relationships between a food product and its related descriptive qualities, components, and processes

FoodOn is an open-source, comprehensive ontology resource composed of term hierarchy facets that cover basic raw food source ingredients, process terms for packaging, cooking and preservation, and an upper-level variety of product type schemes under which food products can be categorized, outlined in Fig. 1.

FoodOn is provided in the Web Ontology Language (OWL) format at the project’s GitHub repository (https://github.com/FoodOntology/foodon), where new term requests and technical support are handled.^7^ The latest version of the resource can also be explored via ontology lookup services like Ontobee (http://ontobee.org), the European Bioinformatics Institute (EMBL-EBI) Ontology Lookup Service (https://www.ebi.ac.uk/ols/), and BioPortal (https://bioportal.bioontology.org).

### The food source hierarchy

In LanguaL the “food source” facet of about 3400 terms describes “the individual plant, animal, or chemical food source from which the food product or its major ingredient is derived.” FoodOn mirrors the organism food source terms closely, with intermediate groups like “stem or spear vegetable”, but moves chemicals (mainly additives) over to a “food component class” to separate them from whole organism references. LanguaL’s food source organisms often have associated species and/or higher level taxonomic identifiers from the Integrated Taxonomic Information System (ITIS) among others. FoodOn preserves LanguaL’s species taxonomic information as database cross-reference annotations. As well, if a FoodOn term’s ITIS reference can also be mapped to an NCBITaxon resource item, then FoodOn uses a ‘has taxonomic identifier’ relation to link the two to facilitate access to NCBI taxonomic and other linked information (e.g., sequence data), as Table 1 illustrates.^8^

**Table 1:** A FoodOn food source term like ‘apple tree food source’ is positioned as a subclass of a common language named food groups like ‘pome fruit plant food source’, and is often qualified by at least one biological taxonomic identifier

Food source term	Logical equivalency	Description
Apple tree as food source	subClassOf 'pome fruit plant food source' and ‘has taxonomic identifier’ some ‘*Malus pumila*’ and ‘has taxonomic identifier’ only ‘*Malus pumila*’	Allows for subclasses of apple tree like honeycrisp (Malus pumila 'Honeycrisp') to be added which identify organism varieties.
European anchovy as food source	subClassOf ‘anchovy food source’ and ‘has taxonomic identifier’ some ‘*Engraulis encrasicolus*’ and ‘has taxonomic identifier’ only ‘*Engraulis encrasicolus*’	‘Anchovy food source’ is a FoodOn class of 13 different species of fish from around the world, one of which is the european anchovy, which has an NCBITaxon species of ‘Engraulis encrasicolus’.
Cricket as food source	subClassOf ‘insect food source’ and ‘has taxonomic identifier’ only (‘*Acheta domesticus*’ or ‘*Gryllus Bimaculatus*’) and ‘has taxonomic identifier’ some (‘*Acheta domesticus*’ or ‘*Gryllus Bimaculatus*’)	Crickets are considered a food source only in the case where instances are one of the given species. Other commonly edible species of cricket can be added to this definition over time.

### The part of plant or animal hierarchy

While some food terms usually refer to a whole edible organism (anchovy, grasshopper), others colloquially refer just to part of an organism (berry, not the bush; apple, not the tree), and some of those parts are not always present or edible in the organism. LanguaL’s “part of plant or animal” facet is defined as “Anatomical part of the plant or animal from which the food product or its major ingredient is derived ...” FoodOn echoes most of LanguaL’s plant and animal part descriptors—both anatomical (arm, organ meat, seed) and fluid (blood, milk)—but reuses existing UBERON and Plant ontology term identifiers for them. This leads to food products like apple being defined as: “‘apple (whole) food product’ SubClassOf: ‘pome fruit’ and ’develops from part of’ some ‘apple tree as food source’”.^9^ Future work may involve detailing the exact parts and stage of life conditions that make a given food bioavailable (for example, an ‘apple’ is only “part of” its tree during the annual fruiting cycle, is only edible when ripe, and needs a proviso that its seeds are lethal if eaten in sufficient quantity).

### Food products and product types

Single or multi-component foods need to be described for food inspection recordkeeping, disease outbreak investigation, food industry supply-chain inventory, and to accommodate dietary restrictions and recipe adjustments. FoodOn design differs from LanguaL in order to achieve this functionality, and these differences are highlighted below.

**Fig. 2:**
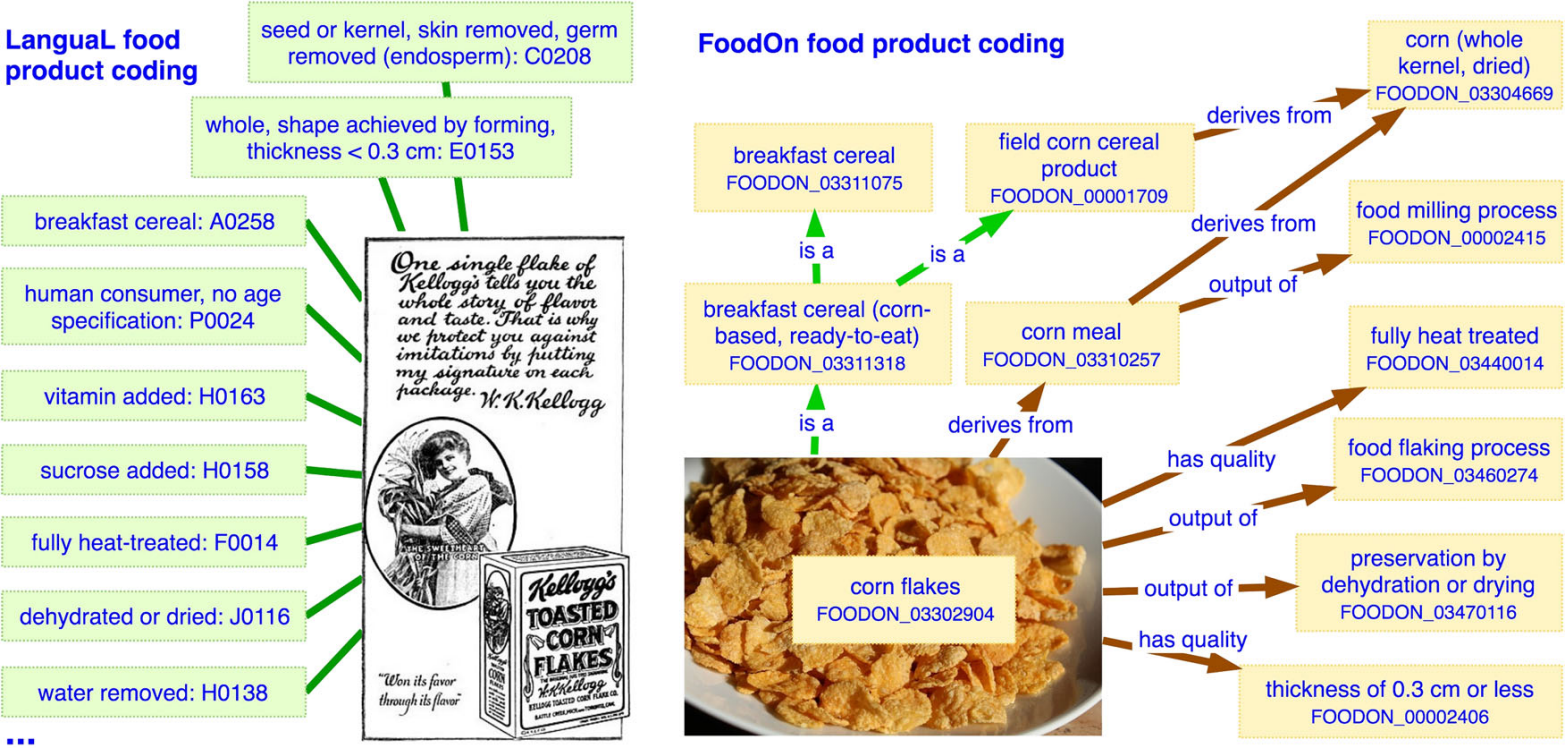
[Corn flakes diagram]. A sample of LanguaL facet terms used to describe a brand name corn flake breakfast cereal, and FoodOn’s corn flakes product representation which uses OWL ontology object properties to link a food product to its components, qualities, and processes

LanguaL’s food product indexing guidelines adequately describe single ingredient foods by allowing one primary food source (facet B) ingredient to be stated which other facets like “cooking method” implicitly reference. LanguaL indexing is typically applied to a database of food items such that “Each food is described by a set of standard, controlled terms chosen from facets characteristic of the nutritional and/or hygienic quality of a food ...", yielding a list of LanguaL term identifiers for each item. For example in LanguaL “corn flakes” would be indexed as a set of facet codes including “A0258 B1379 C0208 E0153 F0014 G0003 H0100 H0138 H0158 H0274 J0116 K0003 M0001 N0001 P0024” as partly shown in Fig. 2, and which can be looked up on the LanguaL website thesaurus page at http://www.langual.org/.^10^

Multiple component foods are more challenging because LanguaL itself does not aspire to be a global food type catalog, and so provides no facility for giving identifiers to component food products. LanguaL suggests curators follow a “Full Ingredient Indexing” protocol in which all ingredients of a product are coded in descending order by weight, but for products like lasagna, one cannot reference components like “lasagna noodle” or “cheese” in the list—only food source items like “durum wheat” are allowed. LanguaL provides one other way to reference other raw ingredients (besides the primary one) by a set of “ingredient added” terms which from an ontology perspective awkwardly duplicate some but not all terms in the “food source” facet.^11^

In a major departure from LanguaL, FoodOn allows food product terms like lasagna noodle (FOODON_03306124) to be defined directly in the ontology, and allows them to reference component products through various relations which do not exist in LanguaL: The “has ingredient” relation applies between two food products, covering the case where a component may no longer be discernable in a final product. “Has part” may be used when a food literally has a part of some other food, unchanged, as in an apple in a caramel apple. The “composed primarily of” relation can replace “part of” if the part is the greatest constituent. “Derives from” is used when a product is transformed by a process in some way from its initial substance, as in applesauce derives from apple. “Develops from part of” is used where a food product is a non-essential part of a food source organism (e.g., zucchini, apple or other fruit). FoodOn has deprecated most of LanguaL’s “ingredient added” hierarchy and instead uses the above relations to reference ingredients. “Output of” indicates that a food product is the product of a given process. “Has quality” holds between a food material and biological, physical, chemical or organoleptic properties which result from corresponding processes. The two approaches to documenting a product are contrasted in Fig. 2.

**Fig. 3:**
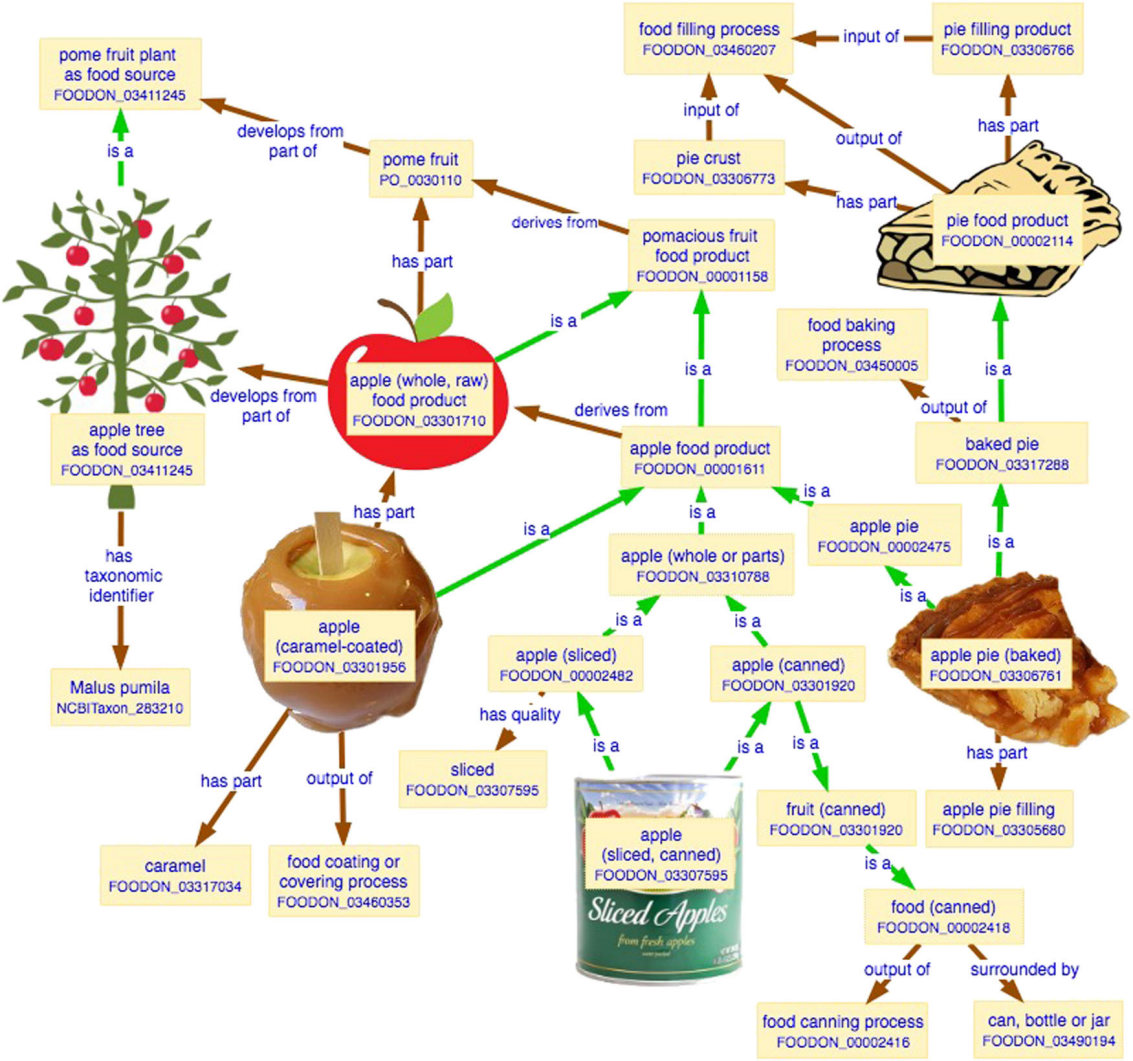
[Apple product diagram]. Overview of apple food products based on “apple (whole) food product”. Products have observable qualities and parts often as a result of the processes that transform them

For food component references to work, FoodOn requires ontology terms and identifiers for all such components. Coverage in this domain has been started by placing food product types (currently numbering 9445 classes) into a “foodon product type” branch, contained in a “foodon_product_import.owl” file. Some of these classes were inherited from the Environment Ontology’s (ENVO) existing sub-domain of food products, while the remainder are from the LanguaL index of FDA’s Scientific Information and Retrieval Exchange Network (SIREN) food database of over 9500 foods which are referenced by FDA regulatory activity documentation, and which anticipate many terms that would otherwise be added piecemeal.^12–14^ Currently, most of the “foodon product type” hierarchy is set explicitly but this will transition to an inferred structure when its growing list of axiomatized products (like sliced canned apples, and baked apple pie, as illustrated in Fig. 3) is sufficiently large.

New FoodOn products can be organized under the foodon product type branch as well as other schemes brought in from LanguaL’s standard product type schemes including the EuroFIR Food Classification and USDA Standard Reference schemes.^5^ FoodOn now has coverage of some asian foods via GitHub requests; other databases (like the LanguaL-indexed French, Greek, and Hungarian ones) could be imported in the future to increase international coverage.

The SIREN food product database does not provide definitions directly, so an ongoing FoodOn task is to populate the imported SIREN terms with appropriate Wikipedia definitions. While consensus on some product definitions may be challenging (for example, should the definition of lasagna expressly allow for cheese substitutes?), FoodOn does want to accommodate the description of more general food categories, as well as food products about which little is known. Conversely, FoodOn avoids too-specific “pre-composed” terms (terms which represent a specific combination of other variables and which verge on recipes). For example, in “apple, raw, without skin, sliced, cooked, microwaved”, removing the cooking method variation allows the class greater applicability. If the cooking method should be preserved in the data at hand, it may be given by a separate field or relationship.

### Food analogs and allergens

It is helpful to link ingredients to substitutes for use in analyses and applications that are sensitive to allergen and other dietary constraints. FoodOn has a “has food substance analog” relation which can connect any two food source items or products, inviting substitution. This symmetric relation allows us to associate natural and synthetic vanilla, but makes no assumption about which side is imitating the other, or the quality of the substitution or appropriate ratio. Pertinent to allergen analysis and food substitution, FoodOn food source terms related to allergic hypersensitivity diseases are being referenced from within the disease ontology (DO).^15^

### Ontology reuse

**Fig. 4:**
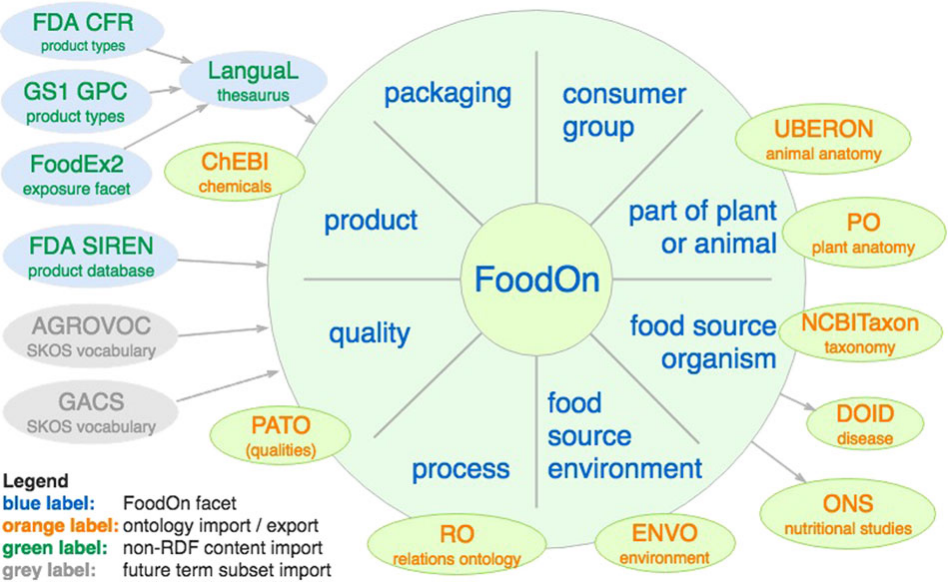
[Foodon component pie-shaped diagram]. FoodOn reuses terms from a number of OBOFoundry.org ontologies as well as LanguaL and SIREN

FoodOn aspires to be a well-documented, actively curated and stable standard, but this depends ultimately on the quality and longevity of its curation model and expert community. As illustrated in Fig. 4, FoodOn’s membership in OBO Foundry enables seamless access to ontologies that cover domains like consumer demographics, agricultural practice, chemical composition and antimicrobials, taxonomy, anatomy, and disease phenotype, which all coexist like mutually-referencing volumes of an encyclopedia.

**Fig. 5:**
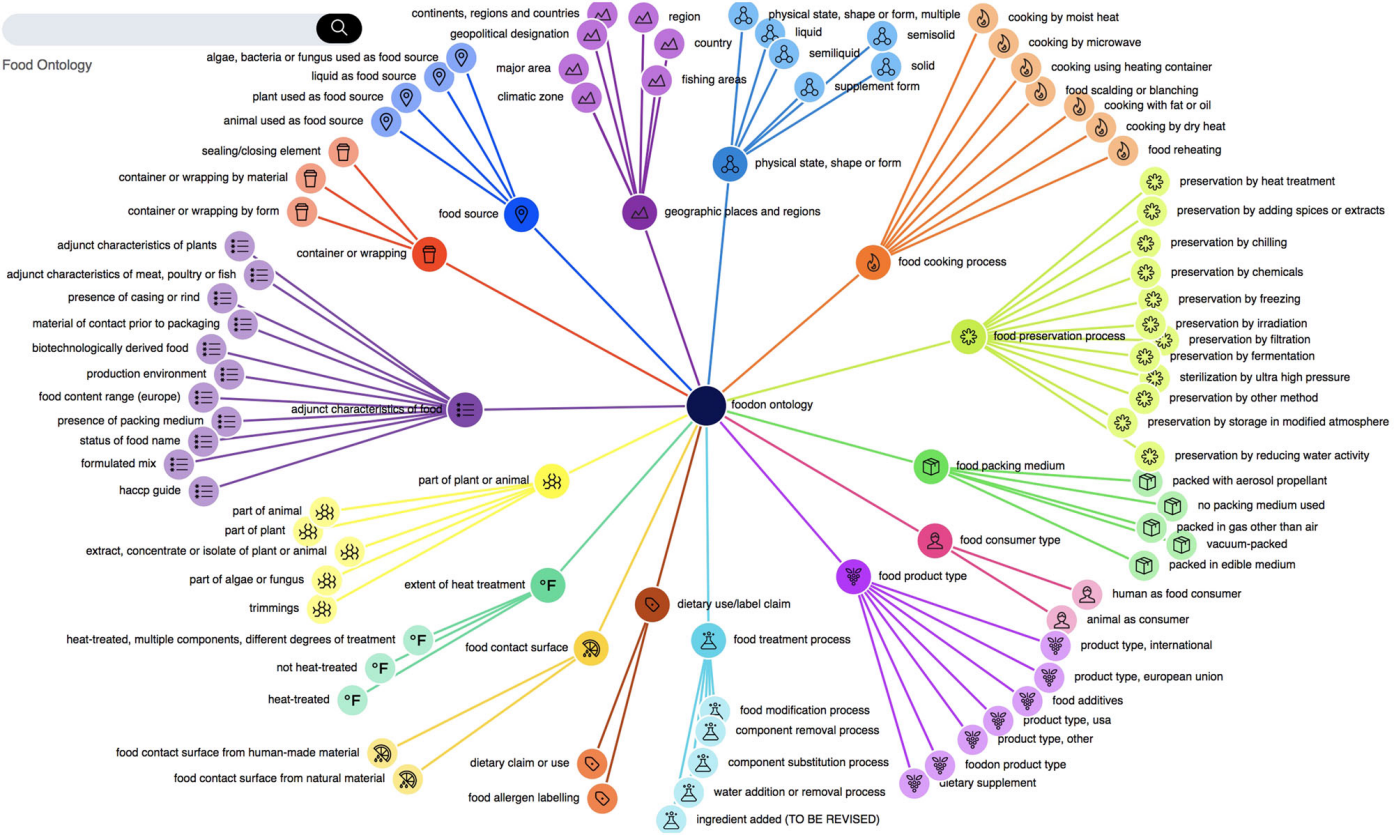
[Subject branch diagram]. A tree visualization of 15 upper-level FoodOn topical branches

**Fig. 6:**
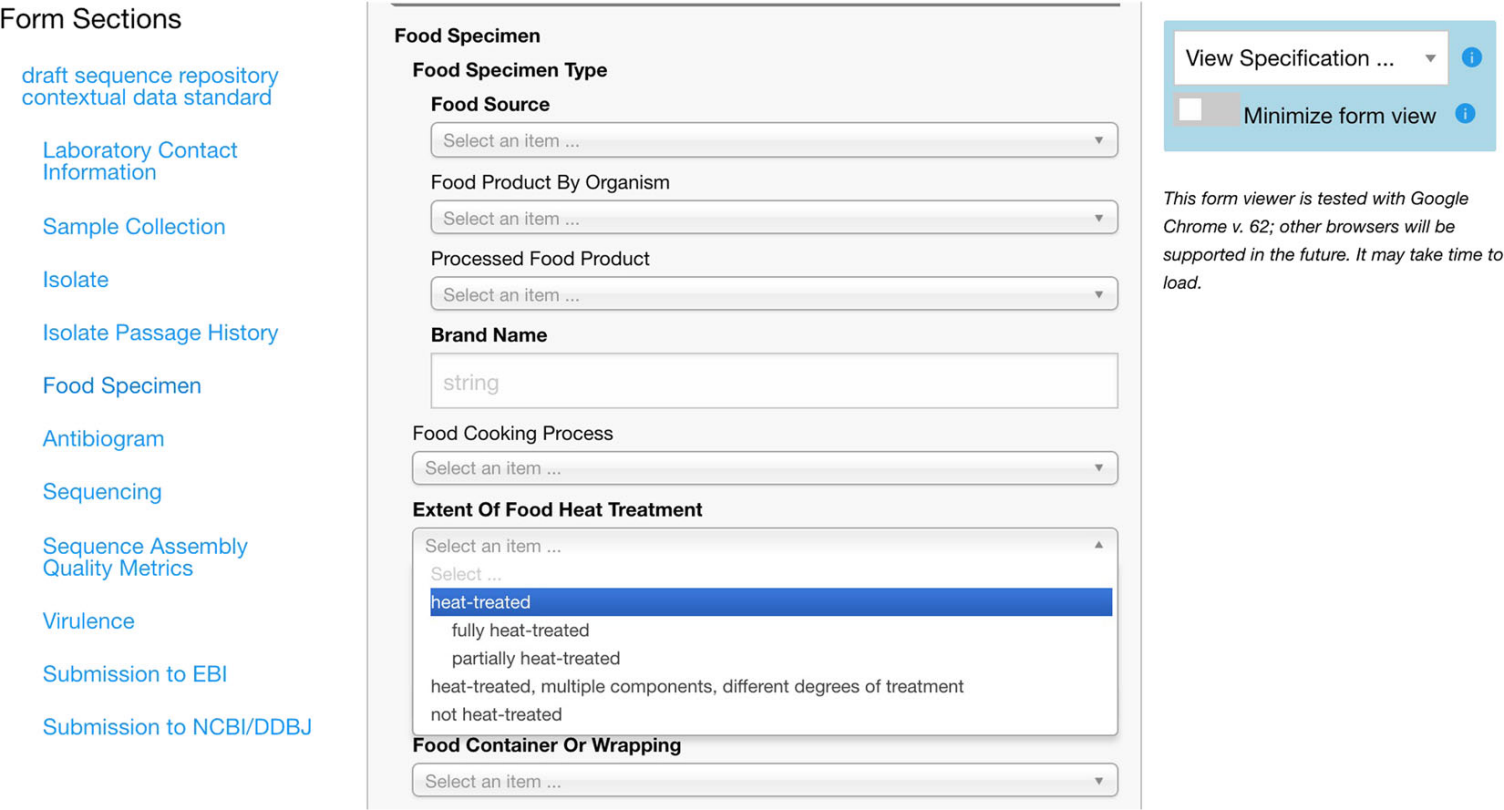
[Form application diagram]. Rendering a FoodOn-driven specification as a web form using the GEEM platform

As shown in Figs 5 and 6, FoodOn aims to cover food products and broad food processing steps, acting as more of a generalist hub that interfaces with more specialist domain ontologies that involve technical food science modeling. This follows the same orthogonal pattern that ENVO has with respect to FoodOn, AGRO, and the CROP ontology among others. FoodOn product hierarchies and relations will continue to expand with new intermediary classes introduced as needed.

OBO Foundry encourages each ontology (with some exceptions) to reuse terms from others where applicable. Reuse of terms allows the effort of providing standardized vocabulary to be shared; so for example, FoodOn has replaced about 600 LanguaL chemicals (e.g., food additives) with ChEBI ontology chemical identifiers.^16^ OBO Foundry ontologies must aspire to a certain overall technical structure, including the upper-level Basic Formal Ontology, curation best practices that involve versioning of ontology files, permanent URLS for terms, and a scheme for annotating deprecated terms’ replacements so that database content can be updated smoothly.^17^

Most relations in Fig. 1 are from the OBO Foundry’s Relation Ontology (RO, http://obofoundry.org/ontology/ro.html) which carry OWL relation domain and codomain restrictions. In FoodOn, these are combined with the upper-level Basic Formal Ontology (BFO) disjoint With axioms, allowing a reasoner like ELK (OWL 2 EL profile) or HermiT (OWL 2 DL profile) to enforce proper reference to processes, qualities, material entities, roles, and information entities in FoodOn. For example, a proposed logical definition for “hen”:

‘hen (food source)’ : 'chicken (food source)' and ('has quality' some 'female organism') and ('has quality' some 'adult organism')

leads to a contradiction because under BFO, the “has quality” relation only permits qualities in its range (right side of the relation). “female organism” and “adult organism” are material entities, a type of BFO “independent continuant” that is disjoint with respect to qualities. In other words, qualities are features of material entities but cannot themselves be material entities. The hen definition can be resolved by stating more directly:

‘hen (food source)’ : 'chicken (food source)' and 'female organism' and 'adult organism'

FoodOn supports reuse in third-party standards via its GitHub repository, allowing users to access and retrieve a particular version or release at any time. However to incorporate such ontology content into agency infrastructure directly often requires a mastery of fairly complex Semantic Web Technology, including knowledge of OWL and the associated SPARQL querying language, as well as the abstractions of an upper-level ontology under which terms are organized.^18^ Various efforts are encouraging ontology reuse without the need for extensive training by providing web portals of customizable spreadsheet or database templates and downloadable specifications, all driven by standardized ontology content.^19,20^ Hsiao Lab is developing a tool that enables marked-up ontology content to be transformed into standards which are provided in both a visual web form and tabular or json version for implementation in data curation and exchange systems^,^.^21^ Figure 5 shows an example FoodOn-driven standard for food specimen contextual data (viewable via Google Chrome at http://genepio.org/geem/form.html#GENEPIO:0002083). Technically this is accomplished using a python script that uses the rdflib module to read an ontology into memory as an RDF graph of triples, and then uses SPARQL to query it and convert it into a JSON representation which the GEEM web interface then renders as HTML forms or downloadable specifications.

## Discussion

Data sharing infrastructure, traceability standards, and ontology communities have come together to made possible a broader vision of a linchpin food vocabulary that can satisfy the need for more complex and standardized datasets. An ontology approach has provided success stories and a way forward for standardizing database content for agencies like the National Center for Biotechnology Information (NCBI), EMBL-EBI, and the UK Department of Health.^22^ Employing FoodOn vocabulary will standardize contractual food references along the farm-to-fork path, enhance research insight and customer satisfaction about more easily comparable food data, speed up traceability of contaminated foods, and ultimately lead to positive economic and human health outcomes. FoodOn consortium partners can draw upon the ontology to standardize vocabulary usage in their interest domains of consumer health, nutrition, food safety and security, and environmental ecology resources, as the following use cases demonstrate.

### Personalized foods and health

The discovery and widespread impact of essential nutrient additives to foods have led to success in reducing nutrient deficiency related diseases, but there has been much slower progress in food-related personalized health insights because of the more complicated relationships between less essential molecules and health phenotypes. “If the scientific, agricultural and food communities are to succeed in improving health, then stakeholders wishing to link food, diet and health information must be able to describe health, and health improvement, through integrated domain ontologies/vocabularies that define the complexity of food, diet, biochemical interactions and phenotypic health.” ^23^ Since FoodOn incorporates vocabularies and axiomatic linkages from across scientific and regulatory domains, stretching from food origins to the final product, it represents the most advanced publicly available ontology for inclusion in personalized food, diet, and health recommender systems. As such, FoodOn is being included as a key part of the IC^3^-FOODS Consortium (http://www.ic-foods.org/) family of Internet of Food (IoF) related ontologies providing clarity about food sources and parts. IC^3^-FOODS brings together industry, government and academic partners to facilitate the transition of isolated information silos to a digitally connected IoF platform.

A second use-case focuses on industry innovation driven by research into consumer convenience and individual food preferences. Devices are being developed that bring contextual awareness to consumer kitchens and food distribution points, for example, touch-screen enabled refrigerators with video cameras that can recognize products on shelves and estimate their shelf-life and timely use in recommended recipes. The industry needs a common low-level food vocabulary to support plug-and-play distribution, shopping, and appliance interconnectivity across competitive lines. FoodOn can supply the raw ingredient and food product type substrate without encroaching on the intellectual property that commercial interests build in higher layers. An open-source, multilingual food vocabulary of basic ingredients and directly derived food products can form the base lingua franca that product research and development efforts reference in proprietary rules or machine learning algorithms that drive food-related software. A commercial recipe phone app could query compatible web-connected storefronts for ingredients (for example, raw celery heart: FOODON_03302922 or raw chicken: FOODON_03301121). Software-driven equipment and consumer appliances like IoT refrigerators or slow cookers could anticipate minimum storage and cooking conditions and durations as those food products are received or produced. Open vocabulary appliances and apps should have a market share advantage by augmenting an underlying standardized and ubiquitous supply chain of input/output food products with food handling intelligence. The alternative is that corporations offer appliances of limited-compatibility using a narrower food vocabulary term set within a vertical of smaller brand product lines and food distribution channels.

### Foodborne pathogen surveillance and investigations

With increasing complexity in food supply chains, foodborne pathogen surveillance and outbreak investigations rely on high-resolution molecular typing results, such as genomic sequencing of pathogens to link related cases and to enable epidemiologists and clinical microbiologists to assess the scope, history, and likely sources of an outbreak.^24^ Currently the traceability component of an investigation - defined as “... the ability to track forward the movement through specified stage(s) of the extended supply chain and trace backward the history, application or location of that which is under consideration...” still depends on a manual paperchase or database lookup of food products and their distribution across disparate systems.^25^ Tracing of contaminated foods is exemplified by the 2011 shiga toxin-producing *Escherichia coli* outbreak from contaminated fenugreek seeds that took months to solve and seriously impacted thousands of people in Europe.^26^ FoodOn can aid in the traceability of such investigations, especially those that occur across borders, by providing standardized identifiers for suspected foods—including dishes specific to a given culture—and their generic ingredients.

Testing for pathogens like *Salmonella* on agricultural sites or in patients, animals, or prepared food, requires a record of the host organism or food product, and/or specimen material (fecal, blood etc.), matrix (swab, wash, etc.) and extraction site. FoodOn includes the required food sources and product categories, but the remaining terms are provided through other ontologies including ones in the domains of anatomy, chemistry, environment, taxonomy, and geography.^16,27^ The Hsiao Lab has created the Genomic Epidemiology Ontology (GenEpiO) as a one-stop-shop specimen-centric vocabulary that incorporates many terms from these ontologies.^28^ FoodOn also provides the vocabulary for tracking simple and complex foods as well as their physical consumption, preparation, or distribution context. Representations of more than 20 contexts that food is sourced from or consumed at—markets, grocers, restaurants, daycare facilities, hospital cafeterias, etc. have been added to ENVO for reuse in FoodOn. This interaction between domain ontologies demonstrates how the federated semantics of OBO Foundry ontologies allows expertise to be pooled for improved interoperation beyond FoodOn’s immediate scope.

### Food traceability

Traceability standards such as the “GS1 standards enabling traceability in the food supply chain” will be enhanced with agronomic and distribution sensor data and encrypted blockchain ledger service providers to monitor food quality and other contractual obligations during a product’s lifecycle.^29–31^ Blockchain technology provides a way for participants in the farm-to-fork relationship—farmers, processors, distributors, and consumers—to access and contribute to a tamper-proof historical record of transactions regarding a food product, yielding food quality and fair cost/payment narratives and easier counterfeit detection. Genomic or other testing can attach a taxonomic species to a given food product sample, and FoodOn’s growing list of ITIS or NCBITaxon identifiers can then be compared to indicate the product label’s veracity. A fraud investigation traceback to some point of deception in the supply chain would then be facilitated by a blockchain or other type of secure ledger. Public health and regulatory agencies and manufacturers seeking to pinpoint a necessary food batch recall period or to halt transmission of the contaminated product will be able to utilize this technology securely and confidentially. As well, the structuring of blockchain transaction content with standards like the GS1 Traceability for Fresh Fruits and Vegetables Implementation Guide, and FoodOn’s extensive list of underlying food products could have a ripple effect in standardizing the food vocabulary of 3rd party systems that contribute to the blockchain ledger.

### Food webs and sustainability

Human food systems - technologically enhanced extensions of a planetary food web - are major components of global ecological processes and systems spanning local to biospheric scales.^32–34^ Their system dynamics massively impact and control human interaction with planetary boundaries.^35–39^ At a global scale, FoodOn and other integrated semantic vocabularies are key tools in monitoring flows of resources and wastes between anthropogenic and natural systems, as well as representing known impacts of the human food system on biodiversity, ecosystem services, and biosphere integrity. FoodOn is designed to be interoperable with ontologies focused on ecological knowledge (ENVO, PCO, ECOCORE), agricultural and agronomic practice (AGRO), and sustainable development (SDGIO), and has included their developers in its founding consortium.^40–43^ This collaboration weaves the semantics of human-centric food systems into a wider ecosystemic and development-focused context. A coordinated curation effort has already ensured that FoodOn’s top-level semantics are synchronized with those used by ontologies adopted by the CGIAR, Bioversity International (e.g., AGRO), and UN Environment (e.g., SDGIO, ENVO), while more fine-grained content is partitioned among these ontologies according to domain relevance. Developing FoodOn within this context will embed data, information, and knowledge about food in a coherently evolving semantic layer connecting multiple stakeholders and leading to improved monitoring technologies.

### Recipe and nutrition analysis

A further area of application for FoodOn is the study of recipes and food preferences, including the link of recipes to nutrition, chemical exposure, food–drug interactions, and distribution and evolution of food preferences. Knowledge about food composition in different geographic and cultural regions is contained in recipes. FoodOn provides standard identifiers for recipe ingredients, thus disambiguating recipe terms and representing them using standardized classes. Nutritional information for recipes can then be estimated from nutritional information available for ingredients. The integration of FoodOn with ontologies of species and anatomical parts can further be used to link food to chemicals contained therein and provide information about chemical exposure, toxicity, and possible food–drug interactions. For example, decomposing recipes in food components, such as chemical compounds, species, and plant structures, can be used to establish a link to phytochemical databases from which information about potentially harmful chemical substances can be obtained. Understanding the chemical composition of foods is important not only to understand nutrition, but also for evaluating risk of toxicity and to reveal potential food–drug interactions.^44–46^ Furthermore, ingredients of recipes can reveal cultural food preferences and possibly provide a link to disease incidence and prevalence within particular populations.^47^

### Future work

A number of the imported facet terms are being formalized using OWL to take advantage of automated reasoning and enable reuse of established ontologies in related fields. FoodOn’s taxonomic classification structure will be developed to support intuitive classification of foods and their facets while maintaining consistency with related ontologies. For example, the “Part of Plant or Animal” facet in FoodOn contains a “meat part” branch that has “skeletal meat part” and “organ meat” categories. Specific organs, e.g., heart, liver, have been mapped to the Uber anatomy ontology (UBERON).^27^ Remaining issues include how to incorporate terms such as blood and marrow, which are not organs anatomically yet should be closely related to “organ meat”. Another issue is that a number of LanguaL’s facets contain terms that can be further defined using a mixture of new classes and existing classes from other ontologies, in particular from the Phenotype And Trait Ontology (PATO).^48^ For example, in LanguaL’s “physical state, shape or form” facet, terms such as 'liquid, low viscosity, with small particles' are partially defined using PATO qualities like 'decreased viscosity', but await a logical resolution for phrases such as “small particles” and “small pieces”. Some FoodOn terms will likely be transferred to other ontologies, for example, since “sliced” is a quality of a material it would fit PATO’s domain. This work will enable existing LanguaL-indexed food databases to be described with more granular components, and will enable an automated reasoner to recognize appropriate members of a food product type and possible component foods.

While FoodOn provides an explicit and growing food product knowledge base for reasoners, there are many areas still to develop or refine. A scheme is needed for natural toxins (for example in cassava) and the food preparation steps required to remove them. FoodOn should also express a maturity or ripeness scheme from INFOODS, which “... can be considered at two states, at the time of harvest/slaughter or at the time of consumption.”, and could cover vocabulary that describes shelf-life and use-by clauses.^2^ FoodOn would benefit from AGROVOC multilingual labels for shared terms, and from GACS, a vocabulary created as the intersection of AGROVOC and two other large vocabularies.^49^ Courtesy of LanguaL 2017, a mapping of FoodOn terms to the EFSA FoodEx2 Exposure facet has been accomplished to enable greater integration to European foodborne disease reporting, but use cases need to be formulated. Granularity is a problem—for example, FAO lists over 12,000 species of fish so it appears many of these need to be imported and connected to regional cultural usage.^50^ Heritage foods, genetically modified food descriptors, and animal feed terminology also need development.^51^ Incorporating resources that make use of FAO INFOODS program standards for preparing food reference materials - including portion weight and measurement terms—would be a natural next step. The FoodOn project will also explore the viability of a term curation portal, in the spirit of Wikipedia curation, that allows people to suggest definitions or links to applicable definitions, (multilingual) synonyms, taxonomy references, “see also”, and image references for terms, which are then vetted by the core curation team.

Hsiao Lab is the primary curator of FoodOn, but the project’s global ambition depends on attracting new partners directly and through the extended network of IC3-Foods working groups currently being organized. The core design of FoodOn has stabilized, so the consortium can now invite participation of private or public organizations to help steer, curate, and provide feedback on its development in a non-competitive environment. FoodOn is participating in the new Global Open Data for Agriculture and Nutrition (GODAN, GODAN.info) working group discussion on harmonization of agency food classification systems. GODAN is a non-profit consortium funded by NGO and government food and nutrition agencies. From these interactions, the FoodOn project expects a viable long-range development plan to emerge that includes both grant funding stimulated by user uptake, as well as a governance model supported by agency-level participation.

## Methods

The Hsiao Lab initiated a search for a standardized food vocabulary in 2015 to populate a metadata component of the Integrated Rapid Infectious Disease Analysis (IRIDA, www.irida.ca) bioinformatics platform for routine surveillance and outbreak analysis of foodborne and other pathogens. To assess the vocabulary needs of epidemiologists and food inspectors, the Hsiao Lab conducted interviews with British Columbia Centre for Disease Control (BCCDC) epidemiologists and examined existing outbreak interview forms of the BCCDC and the Centre for Disease Control (CDC) in Atlanta, and reviewed research data originating from the Government of Canada Genomics R&D Initiative (GRDI) project on agriculture-sector related genomic sampling of farm and food-related specimens. From this exercise, food concepts that needed to be addressed/harmonized were defined. These include edible plant and animal parts and substances with detailed taxonomic and anatomical information; the capability to describe multi-component foods; and processing terms for food preservation and treatment.

A gap analysis was performed between candidate vocabularies and the vision of an OBO Foundry food ontology. Most existing food vocabularies reviewed had shortcomings - inelegant coding, or less organized or incomplete food component description facets. The European Food Safety Association (EFSA) provides FoodEx2, a controlled vocabulary food term catalog whose food product codes are used in mandatory reporting of European Union foodborne illness outbreaks; however this resource lacked facets that could match a food item by description.^52^ The United Nations Food and Agriculture Organization (FAO) provides AGROVOC, a thesaurus that covers a number of food and agriculture-related domains.^53^ AGROVOC has a contemporary, multilingual, and library-science based format (Simple Knowledge Organization System, SKOS) enabling terms to be linked semantically to broader, narrower, and synonym concepts; however it lacks facet descriptors and axioms, thereby making its content difficult to access computationally (see ref. ^54^ discussing its potential ontology conversion; and ref. ^49^ on its SKOS history). INFOODS offered many descriptors for detailing nutritional components of foods, and it reiterated a few of the same LanguaL facets that FoodOn uses for describing food products. INFOODS more open-ended set of facets allow regional data centers to use free-text data entry in fields that merit categorical values. LanguaL was chosen as the best candidate for conversion into a comprehensive ontology. Its single XML file data structure provided clean terms and codes to import, and represented its terms in a fairly straightforward hierarchic format frequently annotated with synonyms, food source taxonomic information and other curation notes that provide professional guidance about how to apply the terms in food indexing tasks. The LanguaL-indexed US FDA SIREN database of food products, available at http://www.langual.org/langual_indexed_datasets.asp, was used as a basis to build a hierarchy of food product items.

Discussions with other OBO Foundry connected curators and food science researchers lead to the creation of the FoodOn consortium, including IC^3^-FOODS, which will be hosting a hub of food-science related ontologies including FoodOn. The consortium has a broad interest including nutritional analysis and food processing aspects which will require more work to satisfy directly in FoodOn and through complementary ontologies like the uc_Milk ontology.^55^

FoodOn currently draws upon 16 OBO Foundry ontologies, culminating in over 27,000 classes. Creation and revision of LanguaL facet term logical definitions is ongoing. FoodOn is being validated against the Enterobase pathogenic sequence database and GenomeTrackr sample descriptions.^55^ FoodOn provides ingredients for the recent Ontology for Nutritional Studies (ONS) under development for the European Nutritional Phenotype Assessment and Data Sharing Initiative (ENPADASI), and is also part of a draft foodborne pathogen sequence repository standard.^56,57^

## Data Availability

FoodOn is available at https://github.com/FoodOntology/foodon in the main foodon.owl file and imports/ folder files, with development work carried on in the src/ontology/ subfolder. The LanguaL conversion script is located in the /src/ontology/imports/langual folder.
